# Multi-Omics Integration of Lactylation- and PANoptosis-Based Signatures in Lung Adenocarcinoma: Prognostic Stratification and Immune Response

**DOI:** 10.3390/ijms26135999

**Published:** 2025-06-23

**Authors:** Zhenhao Xu, Yisha Huang, Xiuling Yu, Jiajia Xuan, Wanting Liu

**Affiliations:** MOE Key Laboratory of Tumor Molecular Biology and Key Laboratory of Functional Protein Research of Guangdong Higher Education Institutes, Institute of Life and Health Engineering, College of Life Science and Technology, Jinan University, Guangzhou 510632, China; zhenhaoxu@stu2022.jnu.edu.cn (Z.X.); yolololohuang@outlook.com (Y.H.); 15332051603@163.com (X.Y.)

**Keywords:** LUAD, prognosis, lactylation, PANoptosis, machine learning

## Abstract

Lactylation and PANoptosis are emerging modes of tumor progression regulation; however, their interplay and effect on the prognosis for lung adenocarcinoma (LUAD) remain unclear. This research analyzed both bulk and single-cell transcriptomic profiles of LUAD and identified 506 potential markers related to lactylation and PANoptosis. Employing 117 machine learning approaches and 5 LUAD datasets, lactylation and PANoptosis-related signatures (LAPRS) and further predictive nomograms were constructed with 85 prognostic genes. The performance of LAPRS was validated with multifaceted validation, including Kaplan–Meier analysis, time-dependent ROC curves and comparison with 55 existing LUAD models. LAPRS enabled the stratification of LUAD patients into high- and low-risk subgroups. Through additional investigation, high-risk individuals showed elevated genomic alterations, reduced immune infiltration, and poorer immunotherapy response, while low-risk individuals showed better drug sensitivity and a higher tumor mutation burden. Further analysis via 18 models and experimental validation revealed APOL1 as a poor prognostic factor, potentially interacting with the lactylation-related gene VIM through TNF signaling. This research clarifies the mechanistic roles of lactylation and PANoptosis in LUAD and proposes APOL1 as a novel prognostic marker, offering insights for therapeutic stratification.

## 1. Introduction

Lung adenocarcinoma (LUAD) persists as one of the most widespread and deadliest malignancies, representing almost 55% of deaths from lung cancer during the previous ten years [1,2]. The high heterogeneity in patient outcomes poses a major challenge in LUAD management, as most patients rarely attain durable responses to targeted therapies or immune checkpoint inhibitors, exacerbating the clinical burden [3,4]. Patient stratification for precision treatment has emerged as a critical strategy to improve therapeutic efficacy [5]. However, conventional staging systems exhibit significant limitations, accounting for patients at the same stage often experiencing divergent treatment responses and survival outcomes due to variations in the tumor microenvironment and host immunity [6]. Therefore, identifying robust biomarkers to forecast prognosis and immunotherapy responses remains critical. Advances involving in silico as well as microarray-based transcriptome and single-cell RNA sequencing have promoted the identification of robust prognostic biomarkers, facilitating more accurate risk assessment [7,8]. In this context, multi-omics data integration, combined with machine learning algorithms, has evolved into a powerful method for building prognostic models based on expression profiles, facilitating precise patient stratification [9].

Lactylation was recently identified as a post-translational modification (PTM) [10]. Numerous studies have shown how lactylation causes lung cancer to progress. According to Gu et al., lactate stimulates CAF and IL-8 release, which mobilizes M2-type or TAMs (tumor-associated macrophages) and intensifies the state of immunosuppression in the tumor microenvironment [11]. Lactate metabolism is intrinsically linked to glycolytic pathways, collectively driving metabolic reprogramming in tumor cells. Within non-small cell lung cancer, the oncogenic transcription factor ETV4 activates the mTORC1 signaling pathway by stimulating glycolysis and lactate synthesis [12]. Alterations in lactate metabolism also contribute to therapeutic resistance in lung cancer. In lung cancer brain metastasis, one study showed that lactate-mediated metabolic reprogramming promotes tumor chemoresistance by driving cell cycle progression through cyclin B1 overexpression [13]. A mechanistic study in non-small cell lung cancer revealed that lactic acid is involved in the TGF-β1/Snail and TAZ/AP-1 pathway, which induces the upregulation of multidrug resistance-associated protein 1 [14]. Therefore, lactylation-related genes have been increasingly found as a potential marker for diagnosis and prognosis in various human diseases, including sepsis, pancreatic adenocarcinoma, colorectal cancer, and lymphoma [15,16,17,18].

PANoptosis is a newly discovered kind of controlled cell death that incorporates apoptosis, necroptosis, and pyroptosis into a unified innate immune inflammatory response [19]. In cancer research, induction of the PANoptosis pathway has been shown to significantly suppress tumor proliferation across multiple malignancies, including respiratory, gastrointestinal, urogenital, glioblastoma, and breast cancers [20]. Mechanistically, PANoptosis is orchestrated by multiprotein PANoptosome complexes, which coordinate the crosstalk between these distinct cell death modalities [21]. Thirteen human cancer cell lines, including the lung cancer models HOP-92 and H22 6, experienced cell death as a result of TNF-α- and IFN-γ-induced PANoptosis activating CASP3, CASP7, CASP8, GSDMD, GSDME, and MLKL [22]. A recent study demonstrated that FADD inhibition markedly suppressed lung cancer cell proliferation, while its knockdown promoted both apoptosis and pyroptosis, identifying FADD as a gene linked to PANoptosis and a potential cancer treatment target [23]. The prognostic significance of PANoptosis has been explored in current publications, including studies on breast cancer, gastric cancer, colon adenocarcinoma and esophageal cancer [20]. Nevertheless, there is little information available about the role that PANoptosis plays in lung cancer, with limited studies reported to date.

Lactylation-mediated epigenetic reprogramming in macrophages regulates tumor-associated immunity, while PANoptosis drives macrophage-induced tumor cell necrosis, suggesting their functional interplay in the tumor microenvironment [24,25]. In epilepsy, lactate metabolism was perceived to modulate PANoptosis through inflammatory–metabolic crosstalk [26]. Despite their prognostic significance, the lactylation–PANoptosis axis remains poorly characterized in cancer.

In this study, we systematically explored lactylation- and PANoptosis-related prognostic genes utilizing bulk and single-cell omics profiles of LUAD. With machine learning approaches, we constructed a powerful predictive model and nomogram enabling the effective stratification of LUAD patients. The high- and low-risk groups were further investigated in genomic mutational landscape and comprehensive tumor microenvironment immune response analyses, including immune infiltration, immune checkpoint and drug sensitivity. Through integrative multi-omics approaches incorporating protein–protein interaction networks, single-cell co-expression analyses, and computational algorithms, we identified APOL1 as a central hub gene functionally bridging lactylation and PANoptosis pathways. Experimental validation further confirmed APOL1’s mechanistic role. Our findings provide both a clinically relevant prognostic framework and potential therapeutic targets for LUAD management.

## 2. Results

### 2.1. Identification of Hub Module and Genes Related to PANoptosis and Lactylation

**Figure 1:**
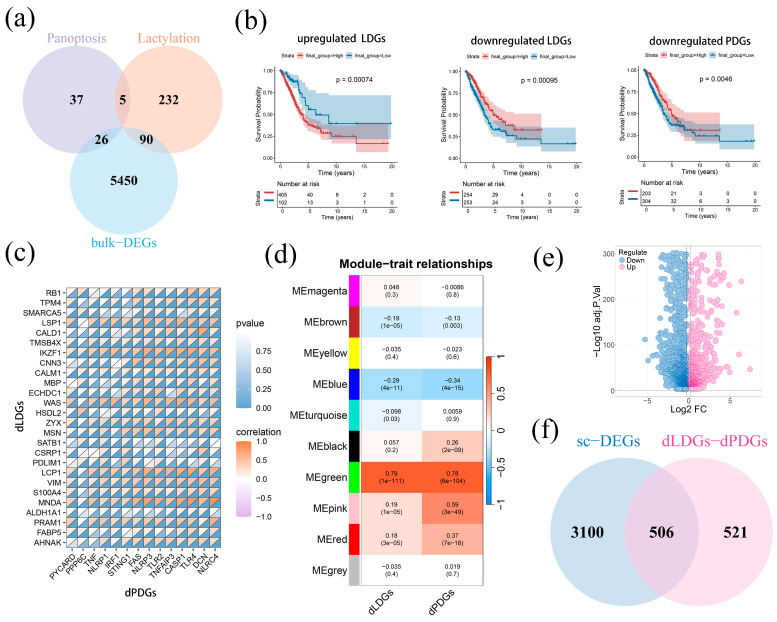
The identification of lactylation and PANoptosis-related differentially expressed genes (LDGs and PDGs) in LUAD: (**a**) the intersected gene between bulk-DEGs, lactylation, and PANoptosis gene sets; (**b**) Kaplan–Meier Survival curves utilizing expression intensity of upregulated LDGs, dLDGs and dPDGs in the TCGA-LUAD dataset; (**c**) dLDGs and dPDGs are positively correlated in the expression level; (**d**) WGCNA analysis of TCGA-LUAD dataset derived nine modules. The green module emerged as the hub module that correlated with dLDGs and dPDGs; (**e**) volcano plots illustrating differential gene expression between tumor and normal groups in scRNA-seq data; and (**f**) the 507 common genes between sc-DEGs and dLDGs-dPDGs.

To first identify the potential prognostic genes related to lactylation and PANoptosis in LUAD, we obtained 5566 bulk-DEGs in bulk transcriptome data TCGA-LUAD (|logFC| > 0.5, *p* < 0.05). The intersection of bulk-DEGs, lactylation and PANoptosis gene sets derived 90 lactylation-related DEGs (LDGs, 64 upregulations and 26 downregulations) and 24 PANoptosis-related DEGs (PDGs, 10 upregulations and 14 downregulations) (Figure 1a). Survival analysis using the minimum *p*-value approach revealed that upregulated LDGs were substantially linked to LUAD patients’ worse prognoses. (*p* < 0.01). In contrast, downregulated LDGs and PDGs consistently demonstrated protective effects, correlating with improved clinical outcomes (Figure 1b). We next observed the complex relationship between downregulated genes in LDGs and PDGs (dLDGs and dPDGs) in co-expression, co-localization, genetic interactions, and predicted associations by constructing a protein interaction network (Figure S1a). Furthermore, dLDGs and dPDGs had a negative correlation in expression (R = −0.43, *p* < 1 × 10^−200^; Figure S1b). However, dLDGs and dPDGs displayed a strong positive correlation (R = 0.84, *p* < 1 × 10^−200^; Figure 1c and Figure S1b), indicating the underlying biological mechanism between lactylation and PANoptosis in LUAD prognosis.

We further applied WGCNA analysis to construct a co-expression network using the aforementioned TCGA-LUAD dataset. Determining the best soft threshold (power = 8, R^2^ > 0.85), we identified a total of nine co-expression modules (minimum 100 genes per module) in which modules characterized by a correlation coefficient > 0.25 were merged (Figure 1d). Among the modules, the green module consisted of 1027 genes that exhibited a strong correlation with dLDGs and dPDGs (R = 0.88 and R = 0.88, *p* < 0.01; Figure S1c). KEGG and GO analyses further showed this lactylation and PANoptosis-related module involved in immune response pathway (Figure S1d).

Exploring the function of the module genes within LUAD cells, scRNA-seq data including 22 samples were processed after quality control and removal of the batch effect (Figure S1e). Subsequently, we derived 30 cell clusters using umap and annotated cell types with marker genes, comprising the mast cell, T cell, B cell, epithelial cell, endothelial cell, fibroblasts and NK cell (Figure S1f,g). We next obtained 3603 DEGs in the scRNA-seq data (sc-DEGs, |logFC| > 0.25, *p* < 0.001, 2713 downregulations and 893 upregulations) and observed the different cell clusters between tumor and normal groups (Figure 1e). Finally, a total of 507 sc-DEGs related to lactylation and PANoptosis wer identified by overlapping 3603 sc-DEGs and the lactylation and PANoptosis-related module.

### 2.2. The Development and Evaluation of LAPRS

**Figure 2:**
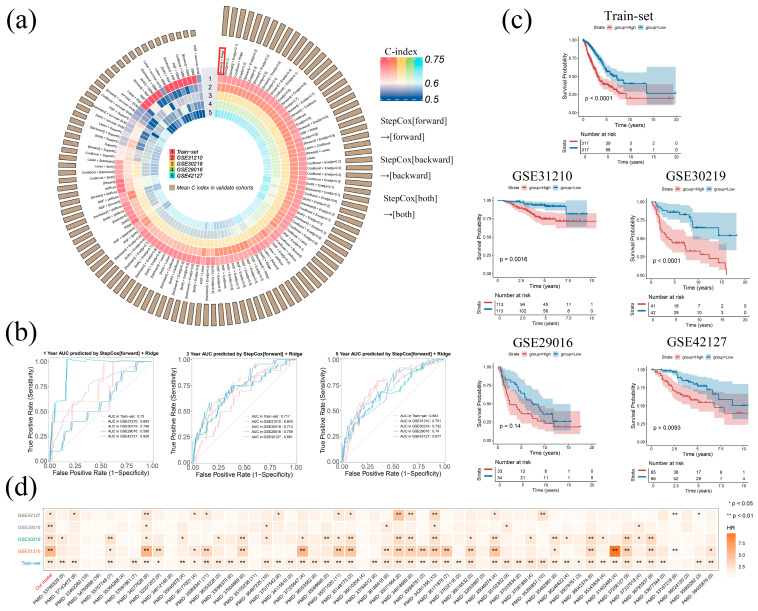
LAPRS was developed using machine learning and exhibited robust performance: (**a**) the performance of 117 models via ten-fold cross-validation, ranked by averaged c-index across validation sets; (**b**) LAPRS exhibit consistent AUC across the training set and validation set in the timeROC curve of 1-, 3- and 5-year OS; (**c**) Kaplan–Meier survival curves of OS according to the LAPRS subgroups across datasets; and (**d**) LAPRS outperformed the 55 published LUAD prognostic models, with the number of genes used in each model indicated in parentheses.

To construct the lactylation and PANoptosis-related signature (LAPRS), we integrated the TCGA-LUAD and GSE50081 datasets as a training set after correcting for batch effects, while GSE31210, GSE30219, GSE29016 and GSE42127 were prepared as external validation sets. This approach enhanced sample heterogeneity and mitigated overfitting risks. To develop a robust prognostic model, we obtained 85 prognostic markers via univariate Cox regression and employed 117 algorithm combinations to train the candidate models. Ranked by averaged C-index calculated in the training set and all validation sets, “StepCox[forward] + Ridge” stood out as the optimal model (Figure 2a).

To assess the model’s robustness and clinical applicability, we evaluated its performance using clinical data and multiple validation metrics. With AUC values of approximately 0.7 in the external validation sets, the ROC analysis demonstrated consistent predictive accuracy, aligning with the training set results (Figure 2b). Next, LUAD patients were stratified into high- and low-risk groups by means of the median risk score derived from the model. Significant survival variations were observed across two groups in all cohorts, with the low-risk group exhibiting better overall survival (OS). This trend was consistent across all datasets except GSE29016 (Figure 2c). Additionally, we systematically compared the ‘StepCox[forward] + Ridge’ model with 55 previously published LUAD prognostic models to further validate the performance of our model. Hazard ratio (HR) analysis demonstrated that our model consistently achieved superior predictive accuracy, exhibiting a higher C-index across all training and validation datasets (Figure 2d and Figure S2a, Table S1). Moreover, our model maintained competitive AUC values, ranking among the top-performing models (Figure S2b). Together, these results underscore the robustness and exceptional prognostic performance of our model in LUAD. Therefore, we designated the ‘StepCox[forward] + Ridge’ model, which comprised 85 signature genes as the lactylation and PANoptosis-related signature (LAPRS).

### 2.3. LAPRS Achieved Better Predictive Capability LUAD Patients with Clinical Information

To determine if LAPRS is LUAD’s independent prognostic effect, univariate and multivariate Cox regression analyses were performed on overall survival (OS) in all sets. The outcomes demonstrated that poorer OS was substantially linked to LAPRS in both univariate and multivariate analyses (*p* < 0.001), confirming LAPRS as an independent factor on LUAD prognosis across all datasets (Figure S3a–e).

**Figure 3:**
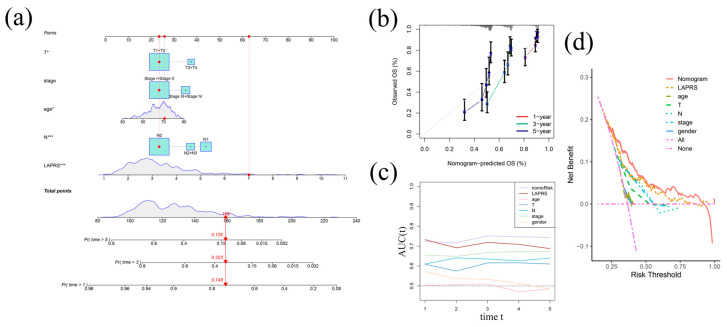
Development and verification of predictive nomogram: (**a**) establishment of the nomogram constructed from the LAPRS and clinical characteristics, including T stage, N stage, and overall stage; (**b**,**c**) calibration curves (**b**) and AUC value (**c**) demonstrating nomogram’s prediction accuracy; and (**d**) decision curve analysis using net benefit evaluation to estimate clinical utility. (* *p* < 0.05, *** *p* < 0.001).

To investigate its clinical applicability and examine the relationship across LAPRS and the pathological and clinical traits of LUAD cases, using clinical phenotypes including age, T stage, N stage, and overall stage, we created a nomogram with the LAPRS risk score. The 1-, 3-, and 5-year OS calibration plots revealed obvious concordance of predicted versus actual survival rates, validating the model’s clinical utility in LUAD prognosis (Figure 3a,b). The inclusion of clinical factors significantly improved the nomogram’s prognostic predictive capability. Both AUC analysis and decision curve analysis (DCA) revealed superior predictive performance compared to individual clinical factors (Figure 3c,d). Collectively, these findings demonstrate that the LAPRS incorporated with age, T stage, N stage, and overall stage can more precisely predict LUAD patient prognosis.

### 2.4. Genomic Alterations in LAPRS Subgroups

**Figure 4:**
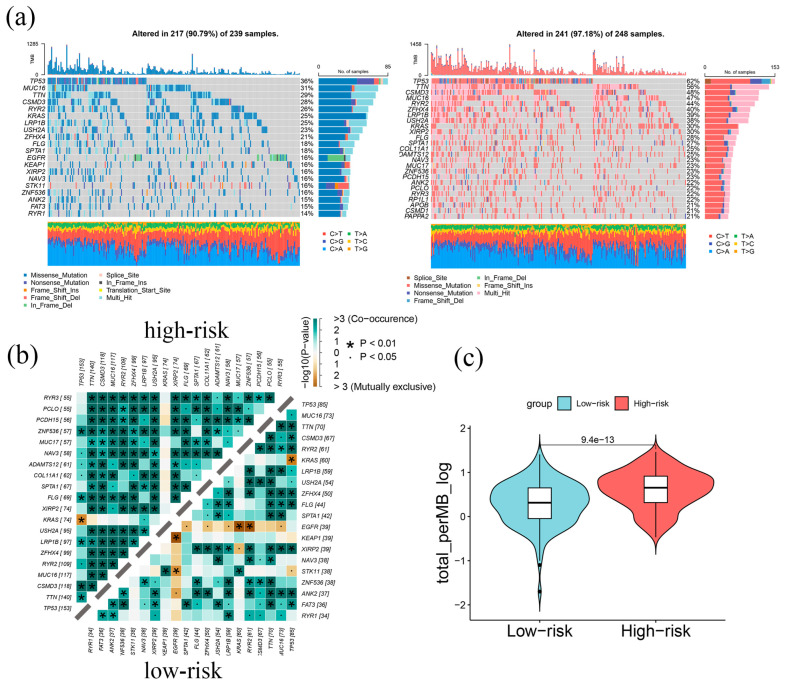
Genomic variation landscape in LAPRS subgroups: (**a**) high-risk patients exhibited higher mutation frequency illustrated in the waterfall plot; (**b**) the comparative analysis described the co-occurrence and association of the top 25 mutated genes in high- and low-risk groups; and (**c**) the variation in tumor mutation burden (TMB) score between high- and low-risk groups.

To investigate genomic alterations across different LAPRS subgroups, we used the TCGA-LUAD dataset to compare the genomic variation landscape across high- and low-risk groups (Figure 4a). Variations among mutational profiles between risk groups were found to be significant by comparative genomic analysis, with the high-risk group exhibiting substantially elevated mutation frequencies (*p* < 0.01). Notably, TP53—a critical tumor suppressor gene regulating cell growth and stress response [27], showed 62% mutation frequency in high-risk patients in contrast to 36% in low-risk patients. Similarly, TTN, which encodes a key protein involved in myofibrillogenesis [28], exhibited a 27% higher mutation rate in high-risk patients, potentially leading to abnormal myofiber development.

The top 25 mutated genes’ co-occurrences, as well as mutually exclusive mutations, were further examined; the results showed clear trends among the subgroups (Figure 4b). While the low-risk patients presented preferential mutual exclusivity, co-occurring mutation frequency was noticeably greater in high-risk patients (Figure 4b). Additionally, tumor mutation burden (TMB) analysis demonstrated a significantly elevated mutation load in the high-risk subgroup in comparison with low-risk counterparts (Figure 4c), further supporting the distinct genomic characteristics between LAPRS subgroups.

### 2.5. LAPRS Associated with Tumor Immune Microenvironment

**Figure 5:**
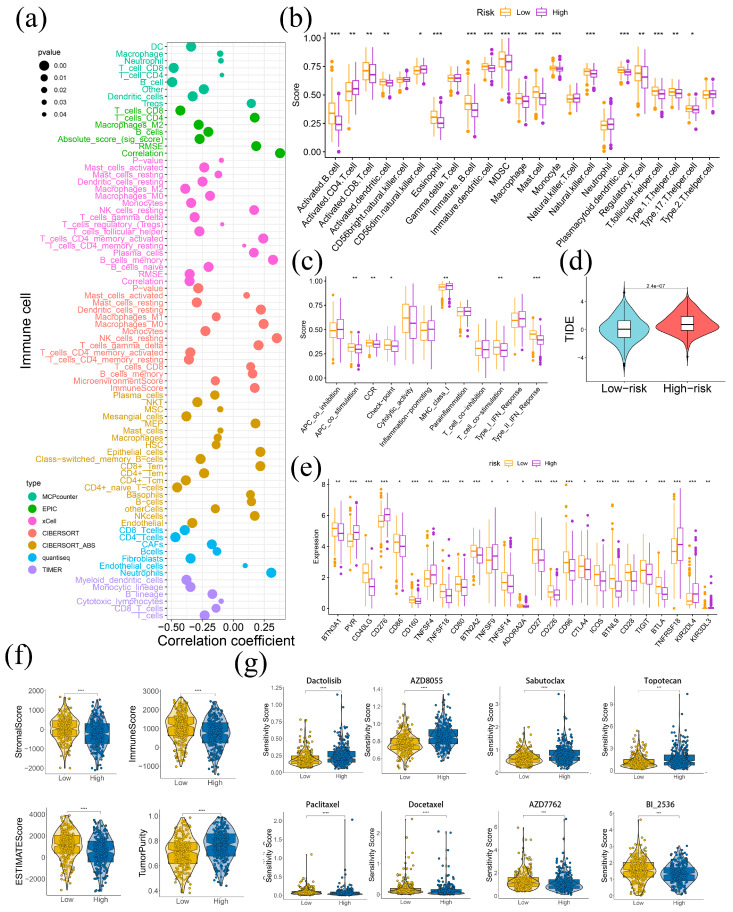
The landscape of the tumor immune-microenvironment with LAPRS subgroups: (**a**) the relationship across immune cells and the LAPRS as determined; (**b**–**e**) the level of immune cells, immune-related function, TIDE score and immune checkpoints; (**f**) the stromal score, immune score, ESTIMAE score, tumor purity; and (**g**) sensitivity scores of eight drugs in different LAPRS subgroups. (* *p* < 0.05, ** *p* < 0.01, *** *p* < 0.001, **** *p* < 0.0001).

To evaluate the LAPRS subgroups’ level of immune infiltration, seven algorithms were employed. A negative correlation for LAPRS was demonstrated by the majority of the immune cell infiltration, as shown by the results (Figure 5a). Specifically, ssGSEA analysis indicated that the infiltration levels of various immune cells, including B cells, dendritic cells, and NK cells, were significantly higher in the low-risk group (Figure 5b). Furthermore, the low-risk group displayed higher scores in multiple gene sets associated with APC co-stimulation, CCR, checkpoint, MHC, T cell co-stimulation, and type II IFN response (Figure 5c). Conversely, LUAD patients with a high LAPRS score were found to have elevated TIDE scores, suggesting a decreased responsiveness to immunotherapy and a greater chance of immune evasion (Figure 5d). Meanwhile, higher expression of immune checkpoints, including CD80(B7), CD86(B70), CTLA4(CD152), ICOS(CD278) and TIGIT were noted in the group at low risk, suggesting increased probability of benefiting from immunotherapy based on immune checkpoint blockage (ICB) (Figure 5e). Using the ESTIMATE algorithm, the low-risk group revealed a significantly higher stromal score, immune score, ESTIMAE score, and lower tumor purity (Figure 5f). Additionally, we explored the drug sensitivity profiles across different LAPRS subgroups. Among these, Dactolisib, AZD8055, Sabutoclax, Topotecan were sensitive to the low-risk group; the low-risk group’s sensitivity score was noticeably lower. Similarly, the high-risk group had a substantially lower sensitivity score in the high LAPRS score-sensitive drugs Paclitaxel, Docetaxel, AZD7762, and BI-2536 (Figure 5g). These drugs may inhibit LUAD progression and serve as potential interventional and preventive measures. Collectively, these findings demonstrated the significance of LAPRS in predicting immunotherapy benefits.

### 2.6. APOL1-Mediated Tumor Progression Is Potentially Mitigated by Lactylation and PANoptosis

**Figure 6:**
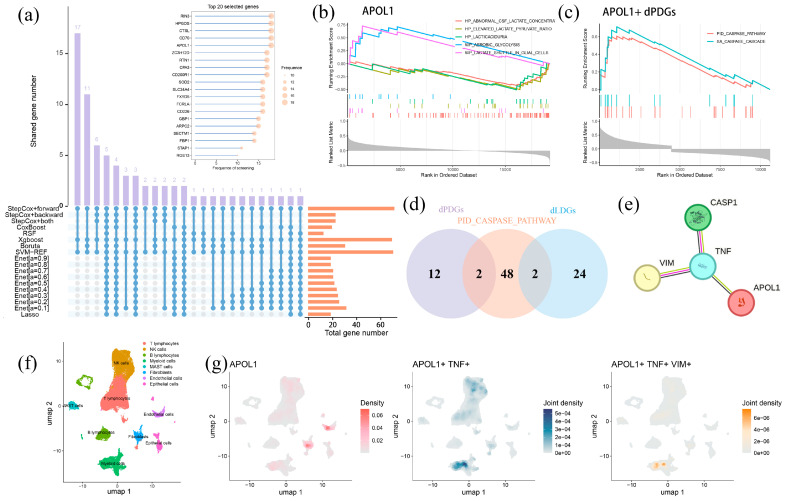
APOL1 emerged as the poor prognostic factor in LUAD engaging in lactylation and PANoptosis: (**a**) top 20 signatures identified by 18 algorithms; (**b**) APOL1 enriched in the top five pathway of the lactylation-related pathway; (**c**) APOL1 enriched two caspase cascade-associated pathways in PANoptosis-related pathways; (**d**) common gene in PID caspase pathway, dLDGs and dPDGs; (**e**) APOL1 communicate with VIM via TNF; (**f**) scRNA-seq analysis displayed the distribution of APOL1 in LUAD; (**g**) the co-expression of APOL1, TNF and VIM.

Finding the essential genes linked to prognosis in lung adenocarcinoma (LUAD), we applied eight computational algorithms and identified the most frequently recurring signatures (Figure 6a). Among the top five candidates, CD70 (TNFRSF7) is a well-established target for immunotherapy, whereas HPGDS (hematopoietic prostaglandin D synthase) and CTSL (cathepsin L) have been widely recognized as prognostic markers in multiple malignancies including LUAD [29,30,31,32]. APOL1 (apolipoprotein L1), an apolipoprotein family member, has been proven to influence tumor proliferation and metastasis in clear cell renal cell carcinoma and pancreatic cancer [33,34]. However, the functions of APOL1 and RIN3 (Ras and Rab Interactor 3) in LUAD remained poorly characterized. Herein, survival analysis revealed that RIN3 had no significant impact on LUAD patients’ OS, whereas high APOL1 expression was consistently associated with lower survival times across multiple cohorts (Figure S4a). Moreover, RIN3 exhibited no differential expression between LAPRS subgroups, while APOL1 was substantially upregulated in the high-risk group (Figure S4c). These surveys implicated APOL1 as a poor prognostic factor. Notably, APOL1 positively correlated with LAPRS scores across multiple datasets (Figure S4b).

To investigate the effect of APOL1 in LUAD, we performed ssGSEA and found that APOL1 suppresses aberrant lactate accumulation while enhancing aerobic glycolysis (Figure 6b). Enrichment analysis linked APOL1 to two caspase cascade-associated pathways, with the PID caspase pathway sharing overlapping genes with dLDGs and dPDGs, suggesting functional crosstalk of lactylation and PANoptosis (Figure 6c,d). Protein interaction analysis demonstrated that APOL1 associates with the lactylation-related gene VIM through TNF, a PANoptosis-related gene (Figure 6e). Furthermore, scRNA-seq analysis showcased the high APOL1 expression in endothelial cells and fibroblasts (Figure 6f). The co-expression of APOL1 and TNF were observed in in NK and T cells, correlating with immune activation and antitumor effects (Figure 6g). APOL1, TNF, and VIM co-expressed in myeloid cells, indicating that PANoptosis–lactylation interplay may counteract APOL1’s tumor-promoting effects by activating caspase cascades (Figure 6g). Meanwhile, drug sensitivity analysis further associated high APOL1 expression with enhanced therapeutic response (Figure S4d). Altogether, these findings show that APOL1’s oncogenic role might be counterbalanced by lactylation–PANoptosis interactions in the tumor microenvironment.

### 2.7. APOL1 Promotes Cell Proliferation and Viability in LUAD

**Figure 7:**
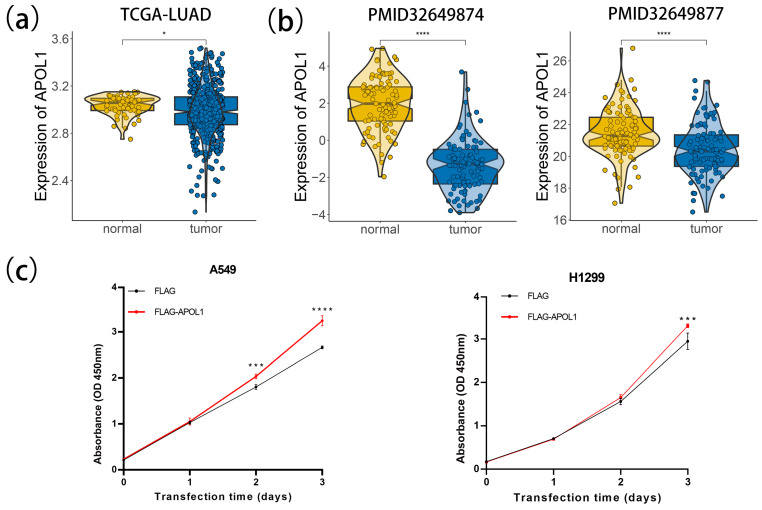
APOL1 promotes the cell proliferation and viability in A549 and H1299: (**a**) differential expression of transcription levels of APOL1 in normal and tumor samples; (**b**) differential expression of APOL1 in proteomics between normal and tumor samples; and (**c**) cell proliferation was assessed by CCK8 assay. (* *p* < 0.05, *** *p* < 0.001, **** *p* < 0.0001).

To explore the role of APOL1 in LUAD, we investigated the expression level of APOL1 in TCGA-LUAD and CPTAC-LUAD; then, we conducted the cell viability assay employing A549 and H1299 lung cancer cell lines. The data analysis showed that APOL1 exhibited low expression in both transcriptomic and proteomics datasets of LUAD (Figure 7a,b). We further overexpressed APOL1 in A549 and H1299, respectively, and found that APOL1 significantly increased the cell proliferation and viability compared with those of the control group (Figure 7c and Figure S5). These results were consistent with the previous analysis suggesting that APOL1 promotes tumor regression in LUAD.

## 3. Discussion

LUAD, a highly aggressive malignancy, demonstrates early invasive potential and marked initial responsiveness to chemotherapy [35,36]. However, therapeutic resistance inevitably develops in nearly all cases, driving disease progression [37]. In this study, we constructed an LAPRS scoring system for LUAD prognosis and unveiled high-and low- risk subgroups reflecting the expression data of 85 genes implicated in lactylation and PANoptosis utilizing both bulk and scRNA-seq data. LAPRS subgroups exhibited contrasting patterns in terms of the genomic alteration landscape, immune infiltration, drug sensitivity, and immune checkpoints. The analysis revealed that low-risk group demonstrates fewer genetic alterations, a higher presence of immune cells, and longer survival times, while the high-risk group presents the converse characteristics. These results proposed LAPRS as an innovative and accurate approach that allows for individualized prognosis and precise treatment guidance for patients with LUAD.

Since their identification as regulatory mechanisms in 2019 [38], lactylation and PANoptosis have emerged as critical players in immune regulation. Protein lactylation, a hallmark of metabolic reprogramming, plays a crucial role in macrophage activation during chronic inflammation [39]. PANoptosis—mediated by PANoptosome complexes—could drives innate immune responses through programmed inflammatory cell death in macrophages [40]. The functional crosstalk between metabolic reprogramming and inflammatory cell death suggests their coordinated interplay in immune regulation. Growing evidence highlights their interconnected roles in modulating inflammatory responses, metabolic adaptation, and disease pathogenesis across multiple pathological contexts. For instance, Gong et al. found that lactylation of cold-inducible RNA-binding protein (CIRP) in macrophages triggered extracellular CIRP release, driving ZBP1-dependent PANoptosis in pulmonary vascular endothelial cells (PVECs) during sepsis-induced acute lung injury [41]. Dichtl et al. revealed that macrophage activation induces histone lactylation (Kla), which inversely correlates with diminished cell death in macrophages harboring mutations in central PANoptosis pathways [42]. In traumatic brain injury, elevated histone lactylation upregulated PSMD14, a proteasomal regulatory component, thereby attenuating neuronal PANoptosis, indicating a potential therapeutic marker for the prognosis of patients [43]. In this study, we observed that lactylation and PANoptosis share common genes. The downregulated lactylation-related genes (dLDGs) and PANoptosis-related genes (dPDGs) in LUAD exhibited a significantly positive correlation, similar regulatory mechanisms, and the same protective prognostic effect. These findings suggest that the proteins involved in lactylation and PANoptosis may work in concert to suppress tumor progression in LUAD. Therefore, incorporating both lactylation- and PANoptosis-related genes into the prognostic model may more comprehensively account for the regulatory mechanisms of lactylation and PANoptosis in LUAD progression. This comprehensive consideration likely leads to a synergistic effect, which may explain why our LAPRS model outperformed the existing 55 LUAD prognostic models.

To explore the hub gene in the LAPRS and its mechanistic role involving lactylation and PANoptosis, we identified APOL1 as a poor prognostic factor that inhibits aberrant lactate accumulation. APOL1 functions in lipid transport, programmed cell death, autophagy, and innate immunity against intracellular pathogens [44]. Recent research revealed that APOL1 contributes to tumor progression. In clear-cell renal cell carcinoma, researchers showed that APOL1 predicts poor prognosis of renal cancer and overexpressed APOL1 promoted tumorigenesis and progression [45]. In pancreatic cancer, APOL1 was found to promote inhibit apoptosis and cell proliferation via the regulation of NOTCH1 signaling, confirming its role as an oncogene [33]. We found that APOL1 exhibited low expression in LUAD and promoted cell proliferation and viability, similar to that reported in previous studies.

Furthermore, our GSEA results showed that APOL1 is involved in the PID CASPASE PATHWAY, which intersects with downregulated PDG TNF and downregulated LDG VIM, indicating the potential interplay of lactylation and PANoptosis in regulating APOL1. Furthermore, APOL1, TNF, and VIM are co-expressed in myeloid cells, while APOL1 and TNF are also co-expressed in T cells and NK cells, indicating the association between APOL1 and the lactylation–PANoptosis axis in regulating tumor progression. Tumor necrosis factor (TNF) is a crucial inflammatory cytokine that can induce PANoptosis [46]. For instance, the synergism of TNF-α and IFN-γ can induce PANoptosis [47]. In this study, PANoptosis may be activated by TNF in response to APOL1. Vimentin (VIM), an intermediate filament protein, was reported to be closely related to tumor invasiveness and prognosis, making it a potential tumor biomarker [48].

Meanwhile, researchers also revealed its significant roles in glycolysis and lactylation [49]. Studies showed that VIM expression levels may also be subject to lactylation, which could influence its function and stability, thereby impacting LUAD progression [50]. In summary, APOL1 not only promotes tumor cell proliferation but also likely participates in inhibiting tumor cell immune evasion in LUAD. The dual roles of APOL1 observed have been validated in other types of tumors. APOL1 may exhibit dual functions that are dependent on concentration or environment, akin to the roles of MAP3K4 and STAT3 in tumors [51,52]. The low basal expression of APOL1 in tumor cells may already be sufficient to fulfill its oncogenic functions, while the downregulation of APOL1 in tumor cells could be a strategy employed by cancer cells to achieve immune evasion. However, the mechanistic role of APOL1 modulated by the lactylation–PANoptosis axis in LUAD requires further experimental exploration.

In summary, leveraging LDGs and PDGs, we successfully developed a prognostic model LAPRS to stratify LUAD patients, and to predict OS and immunotherapy responses. Moreover, we established a predictive nomogram to provide deeper insights into clinical prognoses. Additionally, we identified APOL1 as a poor prognostic factor in LUAD, highlighting its potential as a marker mediating tumor progression. Our findings pave new avenues for elucidating the pathogenesis of LUAD, offering insightful viewpoints for tailored and accurate treatment plans.

## 4. Material and Methods

### 4.1. Data Collection and Statistical Analysis

RNA-seq data for LUAD and corresponding clinicopathological information were sourced from The Cancer Genome Atlas (TCGA) and Gene Expression Omnibus (GEO) database. Samples with missing clinical information were excluded in data cleaning and finally included the following cohorts: TCGA-LUAD (507 tumor samples, 51 normal samples); GSE50081 (127 samples); GSE31210 (226 samples); GSE30219 (85 samples); GSE29016 (67 samples); and GSE42127 (131 samples) (Table S2). All expression data were normalized to transcripts per million (TPM) values and log2 transformed (log2(value + 1)). Genes with TPM < 1 in TCGA-LUAD were filtered out and subsequently analyzed using limma (3.62.2). Differentially expressed genes (bulk-DEGs) were found using adjusted *p* value < 0.05 and |logFC| > 0.5 as the cutoff. The adjusted *p* value was calculated using the Benjamini–Hochberg (BH) method.

Proteomic data were obtained from the studies of Gillette et al. and Xu et al. (Table S3) [53,54]. The 327 Lactylation-associated genes referred to the study conducted by Chang et al. [55], while the 68 PANoptosis-associated genes were identified from Yang et al. [56] and Ferrdb (http://www.zhounan.org/ferrdb/, accessed on 15 February 2025). GeneMANIA (https://genemania.org/, accessed on 18 February 2025) was utilized to construct the functional association network between differentially expressed downregulated Lactylation-associated genes (dLDGs) and downregulated PANoptosis-associated genes (dPDGs) in TCGA-LUAD. The performance of dLDGs and dPDGs in LUAD was evaluated using single sample gene set enrichment analysis (ssGSEA) from “GSEA” (version 2.0.5) with default parameters. The study utilized publicly available data adhering to all relevant access policies and publication guidelines.

### 4.2. Weighted Gene Co-Expression Network Analysis

One method for describing gene association profiles across samples and locating gene clusters with significant co-expression is weighted gene co-expression network analysis (WGCNA). We used the TCGA-LUAD data and the R package ‘WGCNA’ (version 1.7.3). In order to guarantee scale-free network development, the optimal soft threshold (β) was initially selected. Then topological overlap matrix was derived by converting the weighted adjacency matrix, from which dissimilarity was then calculated. Dynamic tree cutting was employed to cluster genes into modules, and the module most strongly correlated with dLDGs and dPDGs was selected for further analysis.

### 4.3. Single-Cell RNA Sequencing Analysis

Samples were collected from GSE131907 (11 samples, tumor; 11 samples, normal) and processed using the ‘Seurat’ package (5.1.0). Variable genes detected in more than 3 cells with expression levels in the range of 200–2500 was selected. Cells with more than 5% mitochondrial gene content were retained for subsequent analysis. The ‘SCTransform’ function was conducted to correct batch effects across samples, followed by the visualization and clustering of cells using uniform manifold approximation and projection (umap). By utilizing marker genes obtained from several cell types in the PanglaoDB database (https://panglaodb.se/index.html, accessed on 11 February 2025), the cell types were labeled. Finally, genes exhibiting differential expression in single cells (sc-DEGs) between tumor and normal groups were identified for downstream analysis.

### 4.4. Development and Validation of Prognostic LAPRS

WGCNA-identified genes in dLDGs and dPDGs-related modules were intersected with sc-DEGs. Genes that intersected were referred to as lactylation and PANoptosis related genes (LAPRGs) in LUAD that differentially expressed in single-cell transcriptome as well as in bulk transcriptome. The prognostic model was built using the ‘MIME’ package (version 0.0.0.9). To find prognostic signatures in LAPRGs, univariate Cox regression was initially used. Then, the TCGA-LUAD and GSE50081 were integrated as training sets after removing the batch effects, while GSE31210, GSE30219, GSE29016 and GSE42127 served as external validation sets. We employed ten traditional machine learning algorithms including random survival forest (RSF), elastic net (Enet), stepwise Cox (StepCox), CoxBoost, partial least squares regression for Cox (plsRcox), generalized boosted regression modeling (GBM), survival support vector machine (survival-SVM), ridge and least absolute shrinkage and selection operator (Lasso) and four possible variable selection filters including Lasso, SetepCox, CoxBoost and RSF. The construction of the models followed these general steps: (1) 117 algorithm combinations in all were conducted using training set to fit prognostic models using ten cross-validation; (2) Across training, internal validation, and external validation sets, all predictive models were evaluated using Harrell’s concordance index (C-index), with performance ranked by average C-index; and (3) The optimal algorithm combination, with balancing robustness and clinical relevance, was selected to establish the final lactylation and PANoptosis-related prognostic signature (LAPRS).

To further evaluate the LAPRS, the 85 predictive signatures in the model were first evaluated using univariate Cox regression, with significant variables incorporated into multivariate analysis. The final multivariate Cox regression identified predictive genes and their hazard risk coefficients for clinical outcomes. The minimum *p* value approach was used to identify the ideal LAPRS cutoff, which stratified cases into LAPRS subgroups. Kaplan–Meier survival analysis was implemented for assessing survival results across LAPRS subgroups. Time-dependent receiver operating characteristic (ROC) curves were additionally constructed utilizing the package ‘timeROC’ (version 0.4) to appraise the predictive accuracy of the prognostic model.

### 4.5. Construction and Validation of Predictive Nomogram

To evaluate the model’s prognostic performance further in clinical application, we used the package ‘regplot’ (version 1.1) to develop a predictive nomogram integrating both the LAPRS and relevant clinical features to estimate overall survival (OS) probabilities in 1-, 3-, and 5-year intervals for LUAD patients. The nomogram’s predictive utility was systematically evaluated through multiple validation approaches, as follows: discrimination capacity was examined using ROC analysis and the C-index; calibration curves were applied to verify calibration accuracy; and, in order to evaluate net benefit and assess clinical applicability, decision curve analysis (DCA) was employed.

### 4.6. Analysis of Genomic Variation Between LAPRS Risk Group

The somatic mutation and copy number variation data of LUAD were sourced from TCGA. The package ‘Maftools’ (version 2.14.0) was employed to examine the mutations landscape in both high-risk and low-risk groups of LUAD patients determined by their LAPRS score. The tumor mutation burden (TMB) score was employed to measure the immunotherapy response.

### 4.7. Enrichment Analysis

Single-sample gene set enrichment analysis (ssGSEA) quantifies the enrichment of specific gene sets in individual samples, with scores reflecting their relative upregulation or downregulation. Herein, we derived lactylation-related gene sets by extracting terms associated with ‘Glycolysis,’ ‘Lactate,’ and ‘Lactic acid’ from the C5.GO (Gene Ontology) and C2.CP (Canonical Pathways) collections in the Molecular Signatures Database (MSigDB, https://www.gsea-msigdb.org/gsea/msigdb, accessed on 15 April 2025). Similarly, PANoptosis-related genes were identified by querying ‘Apoptosis,’ ‘Necrosis,’ and ‘Pyroptosis’ from the same collections. ssGSEA was performed on TCGA-LUAD cases to screen out key pathways mediated by core genes. The package ‘clusterProfiler’ (version 4.14.4) was applied to KEGG and GO analysis.

### 4.8. Analysis of Immune Characteristics, Drug Sensitivity, and Immune Checkpoint

To investigate the association across LAPRS and immune cell infiltration in LUAD, the package ‘IOBR’ (version 0.99.0), which incorporates seven advanced algorithms, was applied to quantify immune cell types and evaluate immunological infiltration. The tumor microenvironment scores were computed utilizing the “estimate” package. Meanwhile, the variations in immune cell signatures, immune functions, and cancer hallmark pathways across LAPRS subgroups were evaluated using ssGSEA. Furthermore, we also included the TIDE score (tumor immune dysfunction and exclusion, http://tide.dfci.harvard.edu, accessed on 3 March 2025) to examine the association between LAPRS and immunotherapy response using Spearman’s rank correlation coefficient.

Comparing the drug sensitivity of the various LAPRS groups, the sensitivity score for the LUAD case was predicted by the package ‘oncoPredict’ (version 1.2), utilizing the Genomics of Drug Sensitivity in Cancer database (www.cancerrxgene.org/, accessed on 4 March 2025).

### 4.9. Hub Prognostic Genes Screening

To identify the core variables in prognostic signatures, the transcriptome features of LUAD patients in the training set and corresponding clinicopathological information were analyzed using univariable Cox regression analysis with the package ‘MIME’ (version 0.0.0.9). Herein, eight machine learning algorithms including Lasso, Enet, Boruta, CoxBoost, RSF, eXtreme Gradient Boosting (Xgboost), StepCox as well as Support Vector Machine–Recursive Feature Elimination (SVM–REF) were utilized for identifying the most frequently recurring signatures. Finally, a total of 18 algorithm combinations were conducted to select the top variables as hub genes associated with LUAD prognosis.

### 4.10. Cell Culture and Transfection

From the Chinese Academy of Sciences’ Cell Bank of Type Culture Collection, we acquired cell lines A549 and H1299. Cells were cultured in Dulbecco’s modified Eagle’s medium, with 10% fetal bovine serum. Mycoplasma and other contaminating bacteria were absent from the cells, as demonstrated by both microscopic examination and culturing. The cells were cultivated in a high-glucose medium in a 5% CO_2_ incubator at 37 °C. To achieve APOL1 overexpression, FLAG-tagged empty vectors (control) and FLAG-tagged APOL1 expression plasmids (FLAG-APOL1) were constructed by Ruibiotech (Guangzhou, China, Supplementary Materials S2). A549 and H1299 cells (1 × 10^5^ cells/well) were transfected with the plasmids (2 μg) using Lipofectamine 2000 (Invitrogen, Waltham, MA, USA) in accordance with the manufacturer’s instructions.

### 4.11. Western Blotting

Cells were rinsed with PBS three times; 100 μL of pre-cooled RIPA lysate (Beyotime, Shanghai, China) containing 1 mmol/L PMSF (Beyotime, Shanghai, China) was added to extract the total protein. The lysate was centrifuged at 13,000 r/min at 4 °C for 15 min and the supernatant was collected. Protein concentration was measured using the BCA method (Thermo Fisher, Waltham, MA, USA) and proteins were denatured at 95 °C for 10 min. A total of 30 μg protein was subjected to 10% SDS–PAGE electrophoresis, transferred to a PVDF membrane, and incubated in blocking solution for 1 h. The membrane was then incubated with primary anti-FLAG (1:1000; ABclonal, Wuhan, China) and GAPDH (1:1000; Fdbio, Hangzhou, China) at 4 °C overnight. After washing with TBST, the membrane was incubated with HRP-labeled secondary antibodies (1:5000; Fdbio, Hangzhou, China) at 37 °C for 0.5 h.

### 4.12. Cell Counting Kit 8 (CCK8) Assay

At 48 h post transfection, logarithmic growth phase A549 and H1299 cells were digested and seeded at a density of 5 × 10^3^ cells/well into 96-well culture plates. They were then cultured for 0, 24, 48 and 72 h. Cell viability was assessed using the Cell Counting Kit-8 test (Glpbio, Montclair, NJ, USA). The optical density was measured at 450 nm and analyzed using a microplate reader (Biotek, Winooski, VT, USA). Every 24 h, the medium was changed for every experiment.

### 4.13. Statistical Analysis

R (4.4.2) was used for all statistical analyses. Spearman’s or Pearson’s rank correlation analysis were conducted to analyze the correlations between two continuous variables. The log-rank test (two-sided) was used to compare Kaplan–Meier survival curves, with patient stratification based on the minimal *p*-value approach. Data was statistically analyzed using GraphPad Prism 9 (GraphPad Software, Inc., La Jolla, CA, USA). The mean ± standard deviation represents the experimental outcomes. The t-test was used to compare the two groups. Unless otherwise noted, statistical significance was considered as *p*  <  0.05.

## Data Availability

The following public databases and web resources were utilized in the study: the TCGA database (https://portal.gdc.cancer.gov/, accessed on 27 January 2025); GEO database (https://www.ncbi.nlm.nih.gov/geo/, accessed on 28 January 2025); Ferrdb (http://www.zhounan.org/ferrdb/, accessed on 5 February 2025); STRING database (https://cn.string-db.org/, accessed on 16 February 2025); GeneMANIA (http://genemania.org/, accessed on 18 February 2025); Molecular Signatures Database (MSigDB, https://www.gsea-msigdb.org/gsea/msigdb, accessed on 15 April 2025); TIDE score (Tumor Immune Dysfunction and Exclusion, http://tide.dfci.harvard.edu, accessed on 3 March 2025); and the Genomics of Drug Sensitivity in Cancer database (www.cancerrxgene.org/, accessed on 4 March 2025).

## Supplementary Materials

The following supporting information can be downloaded at: https://www.mdpi.com/article/10.3390/ijms26135999/s1.
