# Supplementary material for: Multi-Omics Integration of Lactylation- and PANoptosis-Based Signatures in Lung Adenocarcinoma: Prognostic Stratification and Immune Response
Source: Int J Mol Sci. 2025 Jun 23;26(13):5999. doi: 10.3390/ijms26135999 (PMC12249540; doi:10.3390/ijms26135999)
Supplement: Supplementary file 1 [file ijms-26-05999-s001.zip › Supplementary materials 1.pdf]

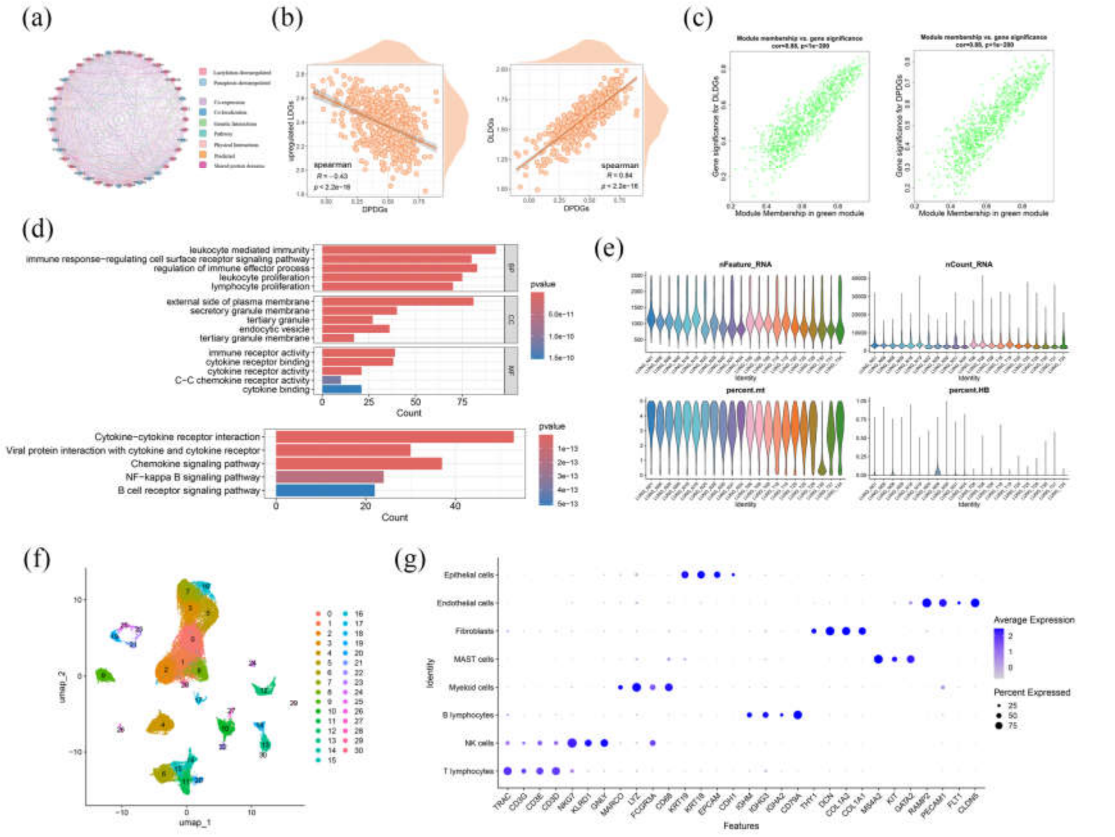

Figure S1. Correlation analysis of A and B in bulk-DEGs and sc-DEGs: (a) Protein–protein interactions among dLGDs and dPGDs based on the GeneMANIA database; (b) Scatter plot showed the correlation analysis of the upregulated LDGs score and dLDGs score with dPDGs score in LUAD patients. (c) Scatter plots showed module membership for each gene within green module. (d) The histograms displaying the results of GO and KEGG enrichment analysis for 1027 genes; (e) The distribution of genes detected, the total number of detected molecules, the percentage of mitochondrial genes and Percent Hemoglobin in each cell for the single-cell data are illustrated; (f) The 30 cell clusters in scRNA-seq data; (g) Expression of marker genes in each cell type.

(a)

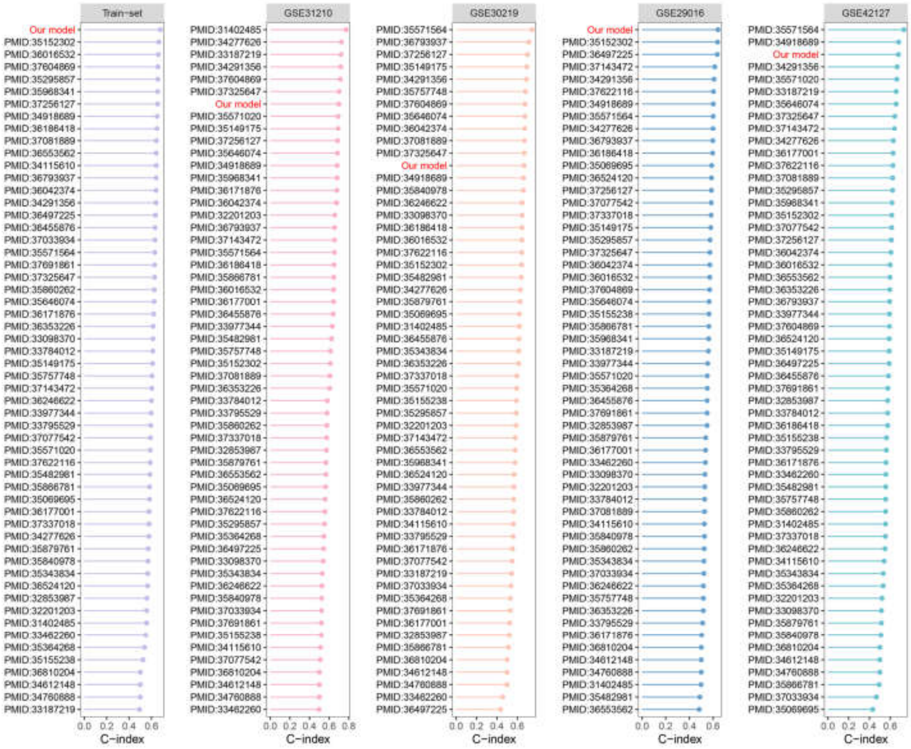

(b)

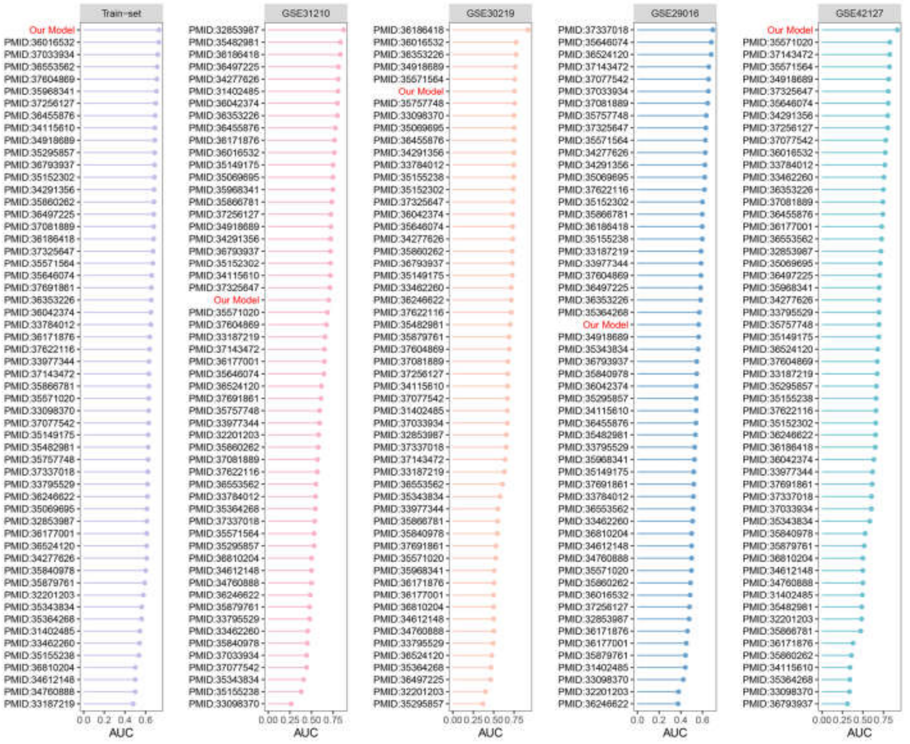

Figure S2. The C-index and first year AUC values of LAPRS and 55 models in the training set and four validation sets: (a) The C-index of LAPRS and 55 models in the training set and four validation sets; (b)

The 1- year AUC values of LAPRS and 55 models in the training set and four validation sets.

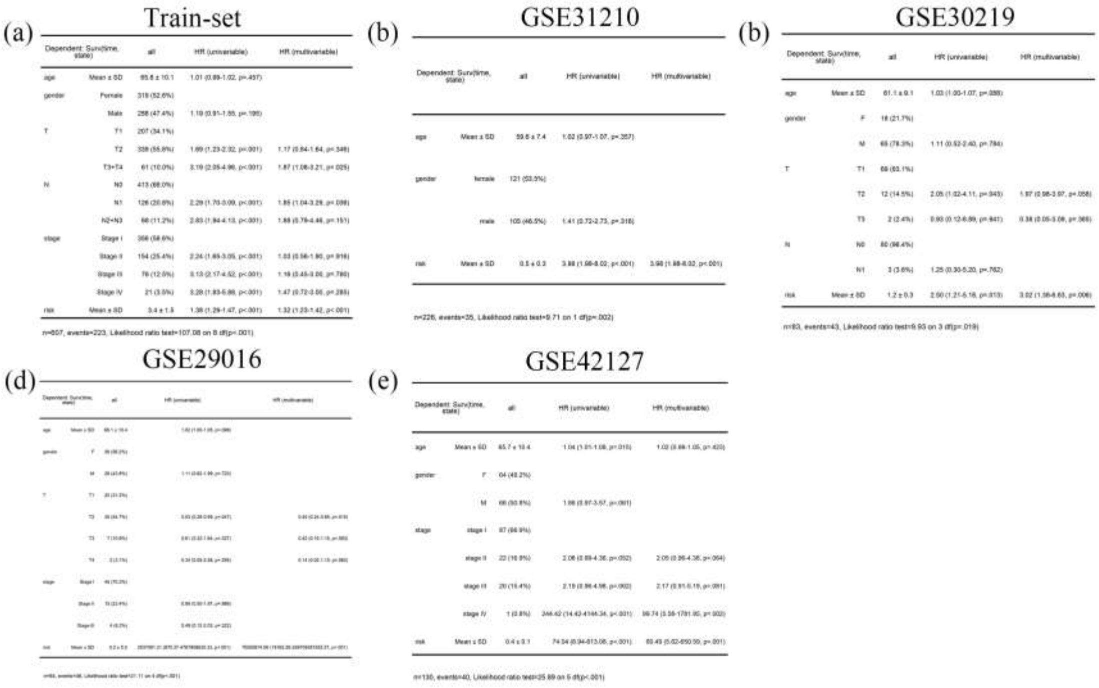

Figure S3. Univariate and multivariate analyses of the training set and four validation sets: (a) Univariate and multivariate analyses of the training set; (b) Univariate and multivariate analyses of four validation sets.

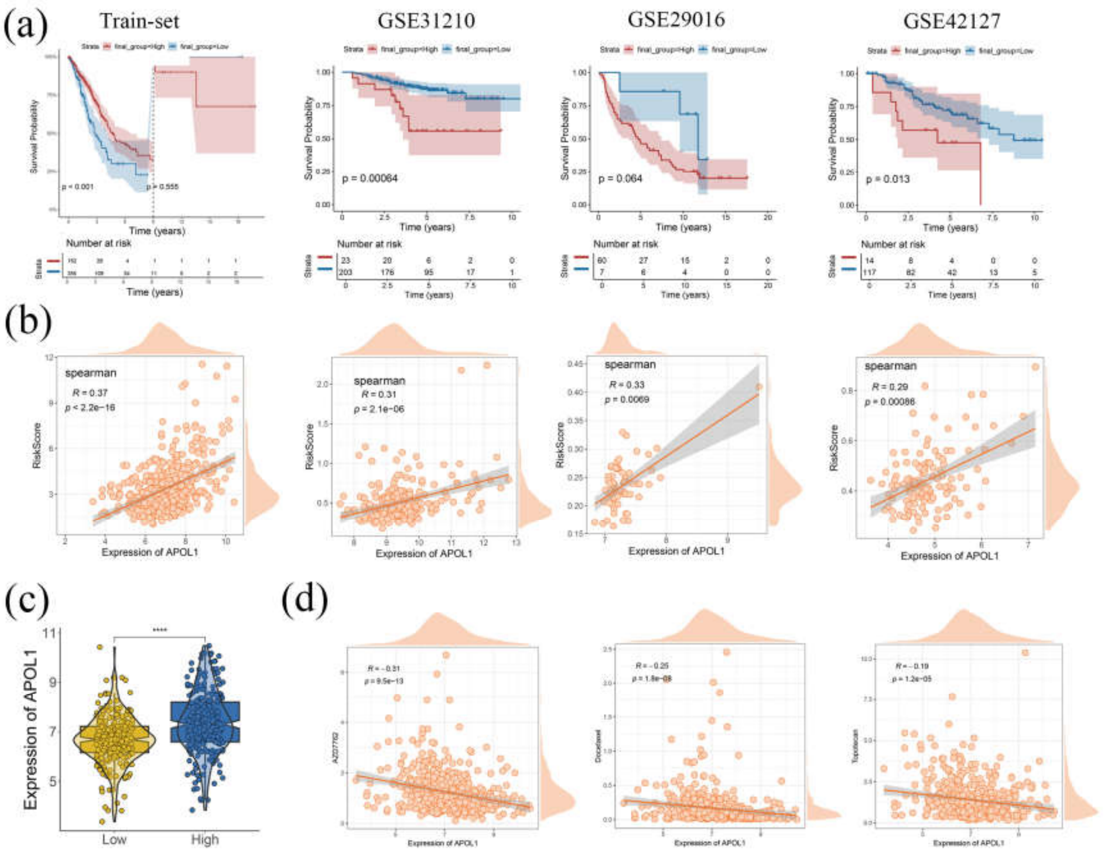

Figure S4. Correlation analysis between APOL1 and LAPRS: (a)-(b) Survival analysis of APOL1 expression in five datasets and its relationship with LAPRS; (c) Boxplot represented the expression of APOL1 in high- and low-risk groups; (d) Scatter plot was created to represent the relationship between APOL1 expression and drug sensitivity.

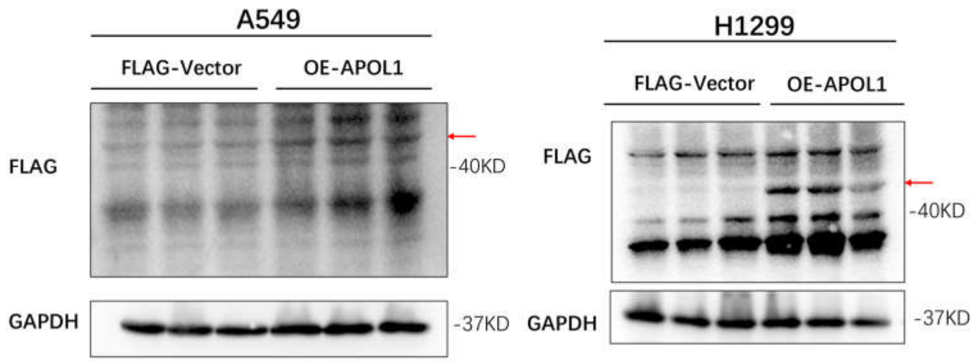

Figure S5. Representative western blot images showing the bands corresponding to APOL1 and GAPDH in A549 and H1299 cell lines transfected with either FLAG-tagged empty vectors (control) or FLAG-tagged APOL1 expression plasmids (FLAG-APOL1).
