# Supplementary figures and images for: Multi-Omics Integration of Lactylation- and PANoptosis-Based Signatures in Lung Adenocarcinoma: Prognostic Stratification and Immune Response
Source: Int J Mol Sci. 2025 Jun 23;26(13):5999. doi: 10.3390/ijms26135999 (PMC12249540; doi:10.3390/ijms26135999)

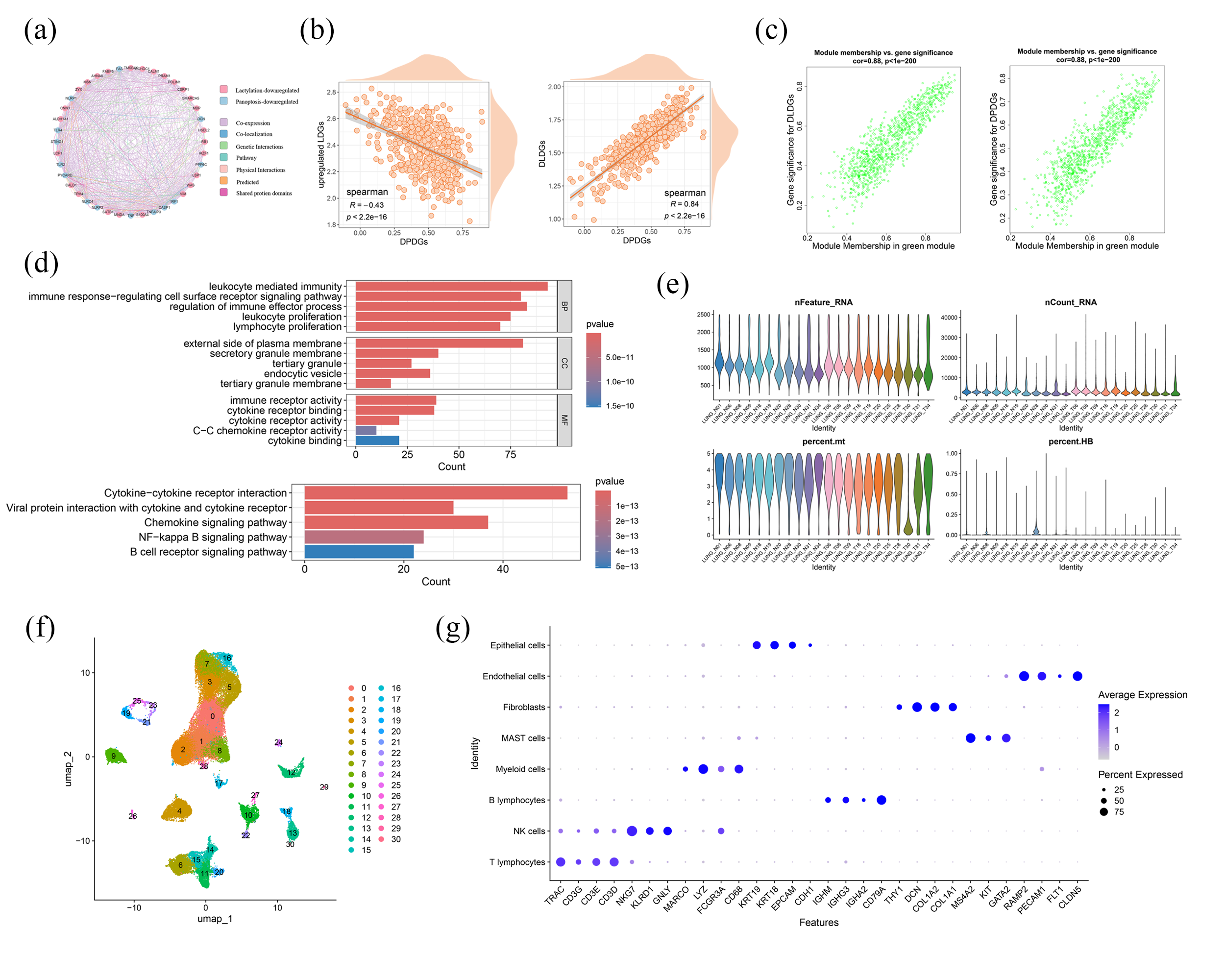

Supplement: Supplementary file 1 [file ijms-26-05999-s001.zip › Figure S1.png]

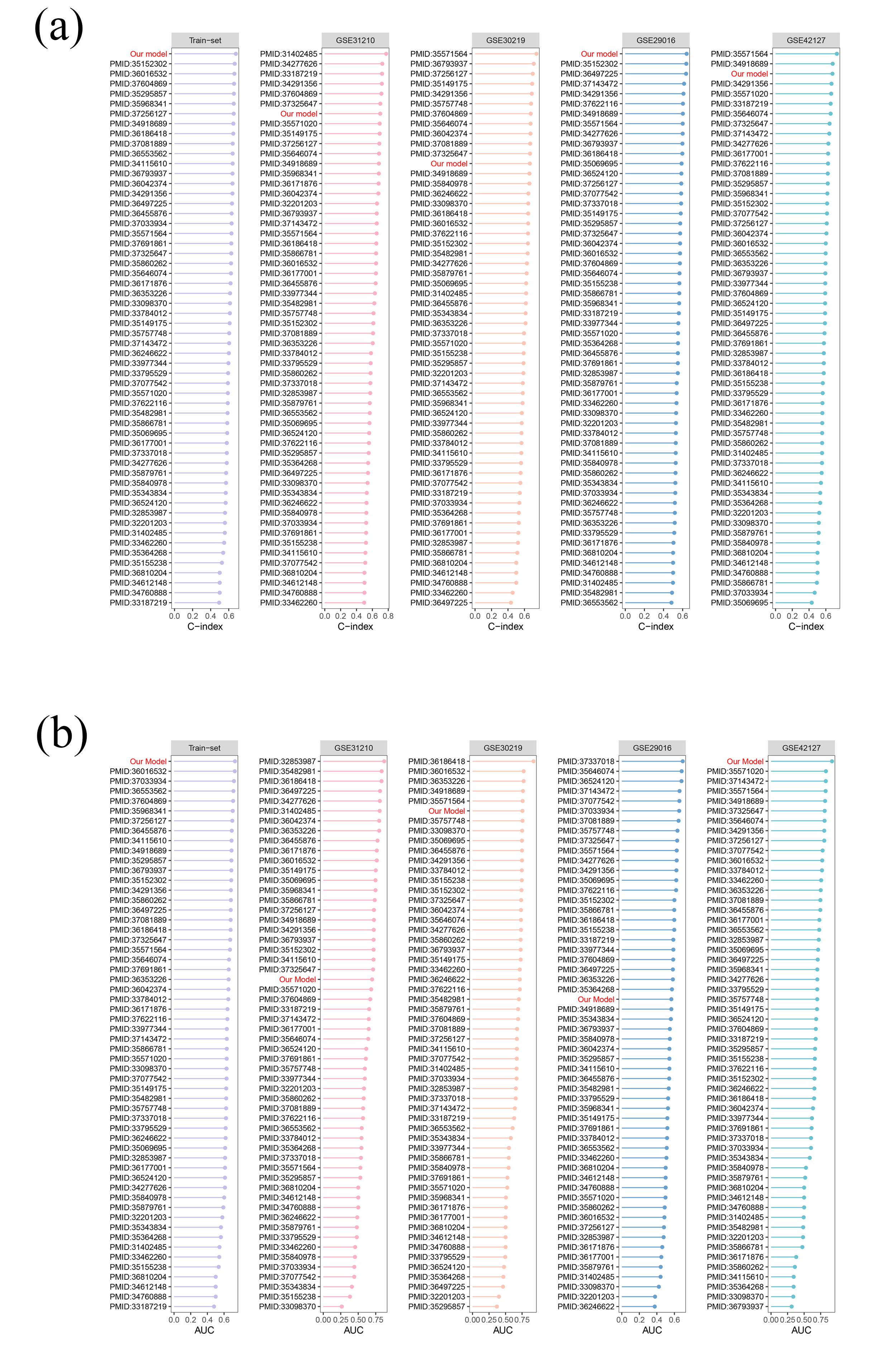

Supplement: Supplementary file 1 [file ijms-26-05999-s001.zip › Figure S2.png]

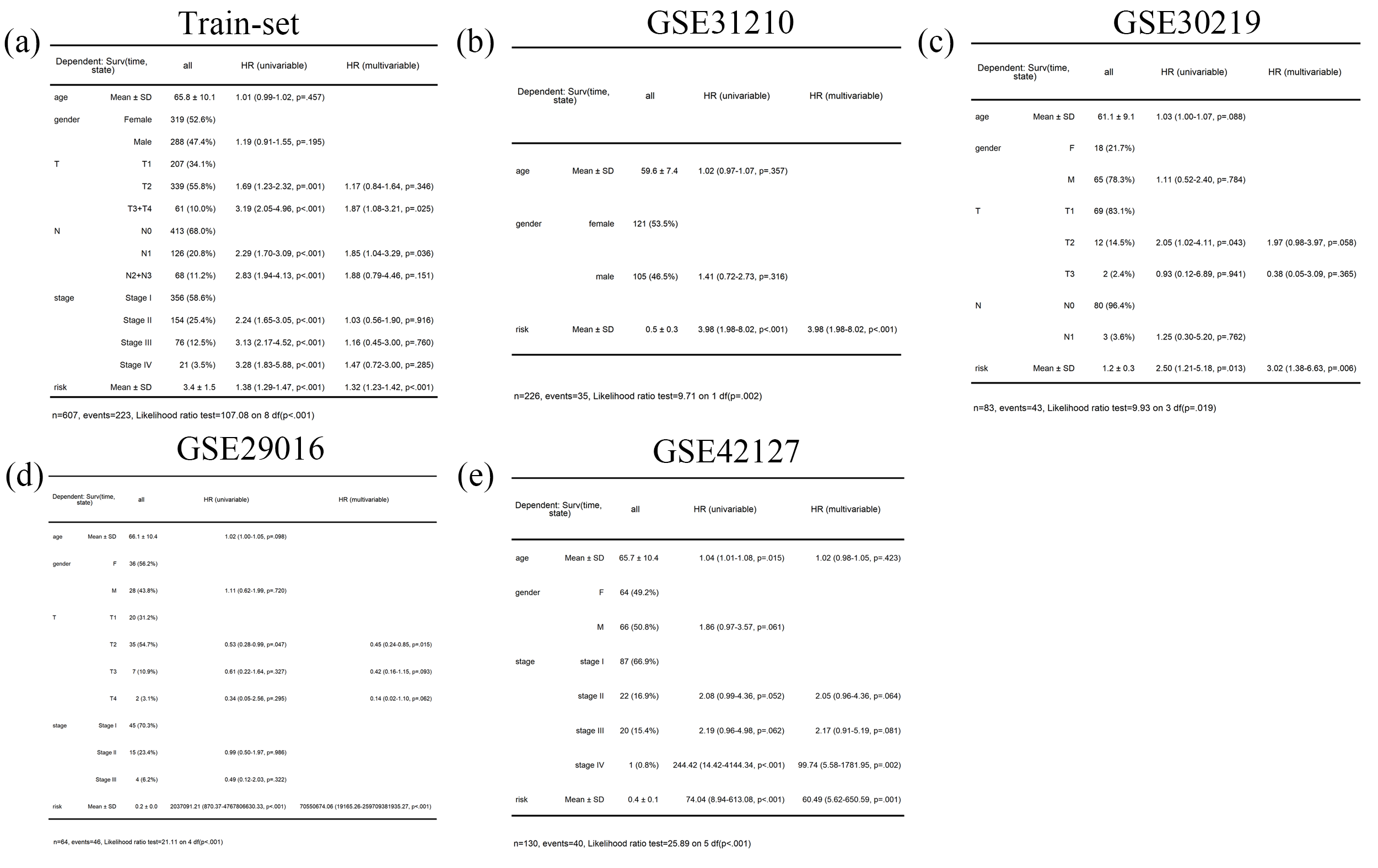

Supplement: Supplementary file 1 [file ijms-26-05999-s001.zip › Figure S3.png]

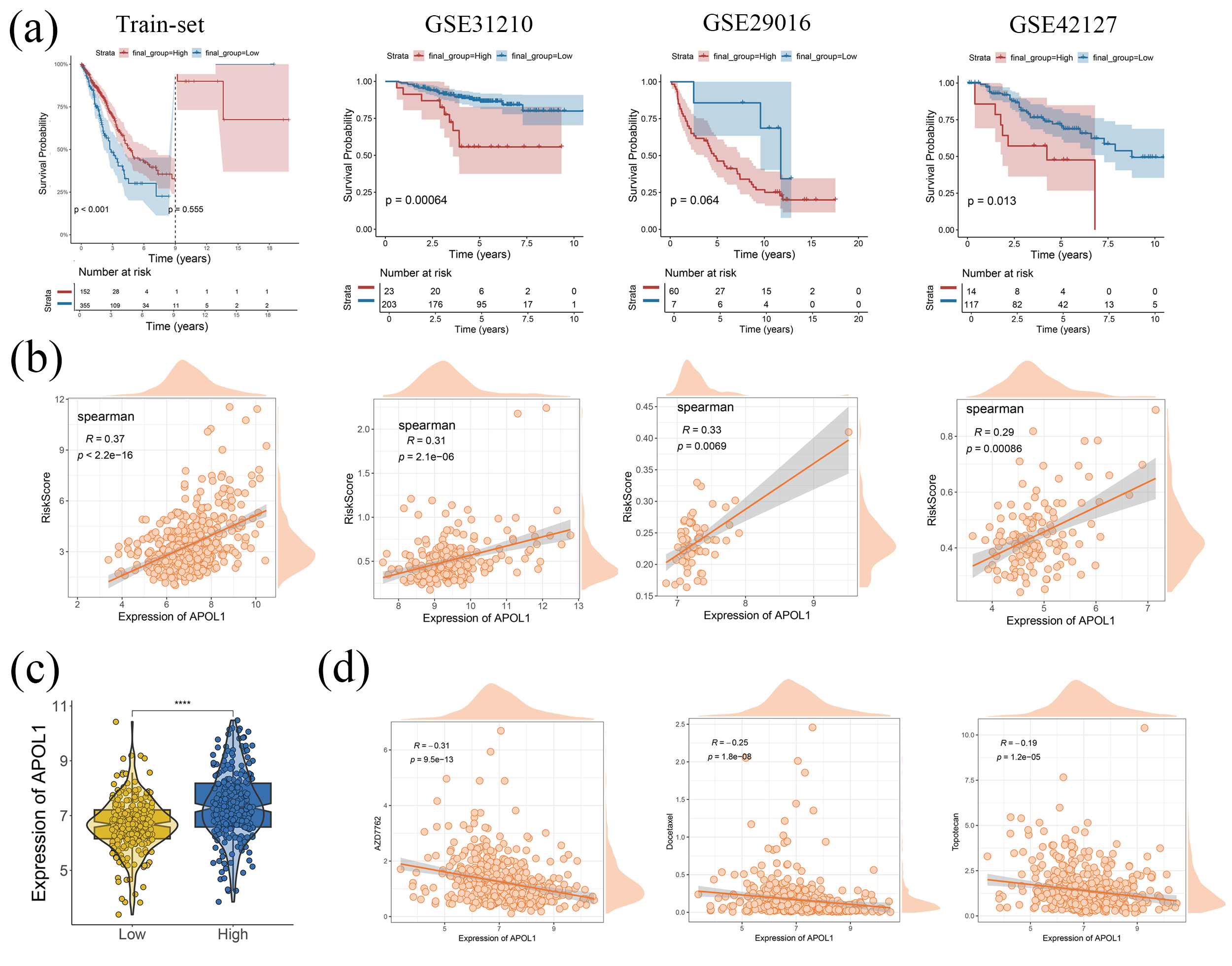

Supplement: Supplementary file 1 [file ijms-26-05999-s001.zip › Figure S4.png]

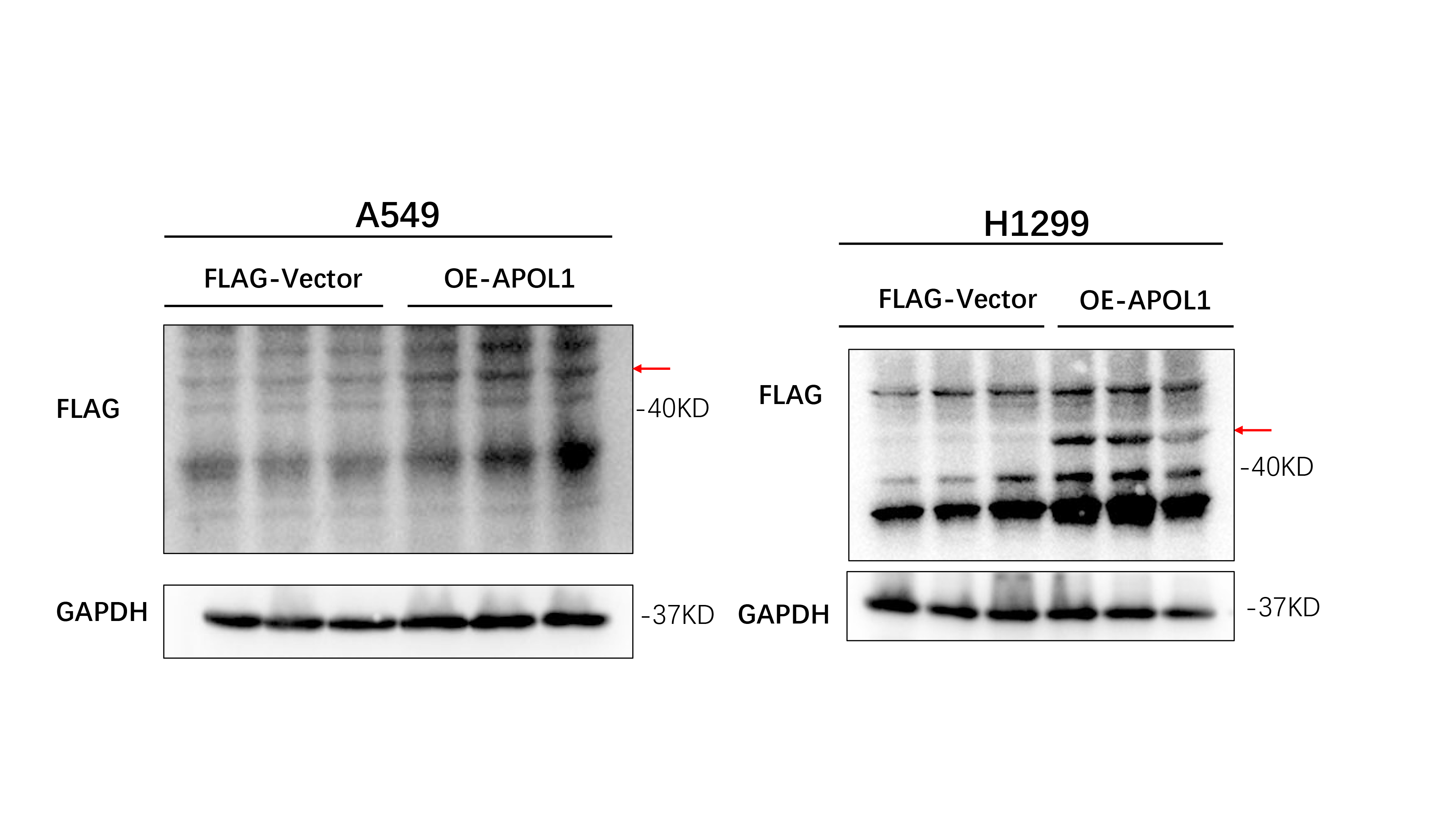

Supplement: Supplementary file 1 [file ijms-26-05999-s001.zip › Figure S5.png]
